# Molecular detection of watermelon virus disease in Liaoning Province, China, and resistance evaluation of nine rootstocks to cucumber green mottle mosaic virus

**DOI:** 10.7717/peerj.21603

**Published:** 2026-08-10

**Authors:** Ming Gu, Bin Zhao, Mingzhu Xu, Mengnan An, Zihao Xia, Zhiping Wang, Yuanhua Wu

**Affiliations:** Department of Plant Protection, Shenyang Agricultural University, Shenyang, Liaoning, China

**Keywords:** Watermelon, Virus detection, CGMMV, Rootstock, Virus resistance

## Abstract

Watermelon diseases have become increasingly severe in Liaoning Province, which is a major watermelon-growing region in China. To investigate the occurrence of watermelon virus diseases and identify the predominant virus types, 330 symptomatic samples from three major producing areas in Liaoning Province (Yingkou City, Xinmin City, Chaoyang City) were tested by seven specific primer pairs using reverse transcription-polymerase chain reaction (RT-PCR). The detection results indicated that the incidence of cucumber green mottle mosaic virus (CGMMV) was 54.85% (181/330), followed by watermelon mosaic virus (WMV) with an incidence of 15.76% (52/330). Considering CGMMV’s predominance and the extensive application of grafting in watermelon production, the disease resistance of nine commercially available rootstocks with diverse *Cucurbitaceae* backgrounds was evaluated. The results showed that all three pumpkin-type rootstocks uniformly exhibited robust resistance to CGMMV, with significantly lower viral accumulation. The bottle gourd rootstock also demonstrated strong resistance to CGMMV. However, wild watermelon rootstocks, due to intraspecific variation and genetic diversity within their germplasm, display a bimodal distribution of high resistance and high susceptibility. These results confirm that CGMMV is the primary watermelon virus in Liaoning Province and highlight the use of pumpkin-type rootstocks as an effective grafting strategy to enhance CGMMV resistance.

## Introduction

Watermelon (*Citrullus lanatus*) is one of the most important horticultural crops worldwide (Guo et al., 2019), with a global cultivation area exceeding three million hectares and an annual production of around 104 million tons (https://www.fao.org/faostat/zh/#data/QCL/visualize). China has been the leading producer, contributing approximately 60.82% to the global watermelon production from 1994 to 2024 (https://www.fao.org/faostat/zh/#data/QCL/visualize). Within China, Liaoning Province has emerged as a major watermelon-producing region due to its favorable climatic conditions and distinctive geographical characteristics. Watermelon production is threatened by a wide range of pathogens, particularly viruses, which can cause substantial reductions in yield and fruit quality (Lecoq & Katis, 2014). A diverse array of viruses has been reported to infect watermelons in China. These include potyviruses, such as watermelon mosaic virus (WMV), zucchini yellow mosaic virus (ZYMV), and papaya ringspot virus (PRSV); cucumoviruses, like cucumber mosaic virus (CMV); tobamoviruses, for example, cucumber green mottle mosaic virus (CGMMV) and tobacco mosaic virus (TMV); orthotospoviruses, such as melon yellow spot virus (MYSV) and watermelon silver mottle virus (WSMoV); poleroviruses, like melon aphid-borne yellows virus (MABYV); comoviruses, such as squash mosaic virus (SqMV); and other viruses including citrullus lanatus cryptic virus (ClCV) and watermelon virus A (WVA).

CGMMV is a positive-sense single-stranded RNA virus belonging to the genus *Tobamovirus* within the family Virgaviridae, and it can systemically infect cucurbit hosts (Antignus et al., 1990). In China, CGMMV is classified as a quarantine pathogen of major concern. The virus was first reported in cucumbers in the United Kingdom in 1935 (Ainsworth, 1935) and has spread globally to Europe, the Middle East, Asia, North America, and Oceania ever since (Dombrovsky, Tran-Nguyen & Jones, 2017). In China, CGMMV was first identified in watermelon and cucumber fields in the northeastern region in 2003, and it was later detected in Liaoning Province in 2005 (Chen et al., 2006). CGMMV infection causes characteristic symptoms in watermelon, such as light or dark green leaf mottling and leaf curling. As the disease progresses, plants exhibit stunted growth, delayed flowering, fruit rot, and severe yield losses (Reingold et al., 2013). Notably, CGMMV infection frequently results in internal fruit decay, significantly reducing market value. Various management strategies have been explored to control CGMMV, among which exogenous boron application has shown promising outcomes. Boron supplementation has been reported to suppress CGMMV infection by regulating nutrient homeostasis (including the uptake and transport of boron (B), nitrogen (N), potassium (K), calcium (Ca), and iron (Fe)) and modulating carbohydrate metabolism, particularly through the regulation of sugar transporter genes (SWEETs) and cell wall-related enzymes (CWINV1, CESA, and COBL7) (Bi et al., 2022). These physiological adjustments can alleviate disease symptoms and partially restore the normal growth of plants.

CGMMV is primarily transmitted through mechanical contact and infected seeds (Dombrovsky, Tran-Nguyen & Jones, 2017). Seed transmission has been documented in at least nine cucurbit species and represents a major pathway for global dissemination. Additional sources of inoculum encompass asymptomatic infected weeds, contaminated soil, irrigation water, nutrient solutions, parasitic plants such as *Cuscuta* spp. (*C. subinclusa*, *C. lupuliformis*, and *C. campestris*), as well as potential insect vectors or carriers including *Apis mellifera*, *Aulacophora foveicollis*, and *Myzus persicae* (Darzi et al., 2018; Qi et al., 2021; Webster & Jones, 2022).

Grafting is widely employed in watermelon cultivation as an effective approach to mitigate both biotic and abiotic stresses (Yavuz et al., 2020; Lu et al., 2022; Jordana et al., 2023; Kappagantu et al., 2023), while also improving yield and fruit quality (Sun et al., 2023). Commonly used rootstocks include *Citrullus lanatus* subspecies (*lanatus*, *C. amarus*, *C. colocynthis*, *Cucurbita maxima*, *C. moschata*), interspecific hybrids (*C. maxima* × *C. moschata*), and *Lagenaria siceraria* (Sakata, Ohara & Sugiyama, 2005; Davis et al., 2008; Tripodi et al., 2020). Among these, interspecific *Cucurbita* hybrids and *L. siceraria* are widely utilized due to their strong contributions to yield improvement and fruit quality (Mashilo et al., 2023). Grafting susceptible watermelon cultivars onto resistant rootstocks is an effective strategy for controlling plant-parasitic nematodes and soil-borne pathogens. For instance, appropriate rootstocks have been shown to enhance resistance to Fusarium wilt caused by *Fusarium oxysporum* f. sp. *niveum* (Yetisir & Sari, 2003; Ling et al., 2013), and wild watermelon rootstocks resistant to southern root-knot nematodes have been developed (Thies et al., 2015a; Thies et al., 2015b; García-Mendívil & Sorribas, 2021). Nevertheless, current research on grafting-mediated disease resistance in watermelon has predominantly concentrated on fungal pathogens, while relatively limited attention has been paid to viral diseases.

Here, we identify CGMMV as the predominant viral pathogen affecting watermelon crops in Liaoning Province. Given that the majority of cucurbit crops are generally sensitive to CGMMV, we conducted a resistance test against CGMMV on nine common cucurbit rootstocks collected from the seed market. These nine rootstock varieties included pumpkin-type rootstocks, bottle gourd rootstocks, and wild watermelon rootstocks, with each type consisting of three varieties or subspecies. By clarifying the differentiation patterns of the resistance of different genera and species of rootstocks to CGMMV, this study can provide a reference for optimizing the selection of rootstock combinations, taking into account both disease resistance and cultivation traits. Moreover, it can offer materials and a theoretical basis for molecular marker-assisted breeding and research on the disease mechanism of cucurbit crops against CGMMV.

## Materials & Methods

### Plant materials

A total of 330 naturally infected watermelon leaf samples showing typical virus-like symptoms, including yellowing, mosaic, chlorosis, and leaf curling, were collected from protected and open-field cultivation areas in 11 villages across Yingkou, Xinmin, and Chaoyang, Liaoning Province, China, between July 2023 and August 2024 (Table S1). Each sample was labeled according to its collection site and transported in insulated foam boxes. Upon arrival, samples were photographed, divided into subsamples, and stored at 4 °C until total RNA extraction.

### Nucleic acid extraction and reverse transcription-polymerase chain reaction

To identify viral pathogens in the surveyed regions, seven major watermelon viruses previously reported in China were selected for detection: CGMMV, CMV, WMV, TMV, ZYMV, MYSV, and WSMoV (Table S2). These viruses were selected based on their reported incidence, economic importance, frequent detection in previous surveys, and ability to induce overlapping symptoms that complicate field diagnosis.

For initial screening, the 330 field samples were grouped into pooled samples, with five individual leaf samples combined into one pooled sample. Total RNA was extracted from each pooled sample using the FreeZol Reagent Total RNA Extraction Kit (Vazyme, Nanjing, China) according to the manufacturer’s instructions. First-strand cDNA was synthesized using the HiScript^®^ III 1st Strand cDNA Synthesis Kit (+gDNA wiper) (Vazyme, Nanjing, China). Reverse transcription-polymerase chain reaction (RT-PCR) was then performed using previously validated virus-specific primers targeting the seven selected viruses (Liu et al., 2019). Polymerase chain reaction (PCR) amplification was carried out in a 20 µL reaction system containing 1 µL cDNA template, 10 µL 2 ×Taq Master Mix, 0.5 µL of each primer (10 µmol L^−1^), and 8 µL RNase-free ddH_2_O. The thermal cycling conditions were as follows: initial denaturation at 94 °C for 30 s; 30 cycles of 98 °C for 10 s, annealing at virus-specific temperatures for 30 s (Table S2), and extension at 72 °C for 15 s; followed by a final extension at 72 °C for 2 min.

For pools that tested positive, the five individual samples within each pool were subsequently subjected to separate RNA extraction and RT-PCR detection using the same protocol to identify the infected individual samples. Final infection rates of each virus were calculated based on the results of individual sample testing.

### Sequence identity and phylogenetic tree analyses

To further confirm the causal viral pathogens, two symptomatic isolates were selected from each of the three major watermelon-producing regions: Yingkou, Xinmin, and Chaoyang. RT-PCR was performed to amplify the full-length genome of CGMMV and the coat protein (CP) coding region of WMV. Specific primers were designed based on reference sequences of CGMMV (GenBank accession number KY040049) and WMV (GenBank accession number KU246036) (Table S2).

PCR amplification was performed in a 50 µL reaction mixture containing 2 µL cDNA template, 25 µL PrimerSTAR Max, 1 µL of each forward and reverse primer (10 µmol L^−1^), and 21 µL RNase-free ddH_2_O. The cycling conditions were as follows: initial denaturation at 94 °C for 5 min; 30 cycles of 98 °C for 10 s, 56 °C for 5 s, and 72 °C for 1 min for CGMMV genomic fragments or 30 s for CGMMV-CP and WMV-CP fragments; followed by a final extension at 72 °C for 10 min. PCR products were visualized on a 1% agarose gel, purified, and cloned into the pMD19-T vector (TaKaRa, Dalian, China). Positive clones were subjected to Sanger sequencing by Sangon Biotech (Shanghai, China). The obtained CGMMV and WMV sequences were compared with available sequences in the NCBI database using BLAST. Multiple sequence alignments were performed using representative CGMMV genome sequences and WMV CP sequences from GenBank. Phylogenetic trees were constructed using the Maximum Likelihood method in MEGA version 11.0 with 1,000 bootstrap replicates (Koichiro, Glen & Sudhir, 2023).

### Plant preparation and Virus inoculation

‘Zhongxu mengbao’ were obtained from Qingdao Zhongxu Seed Industry Co., Ltd (Qingdao, China); ‘Yezhen No. 6’ were obtained from Lanzhou Mirui Agricultural Science and Technology Co., Ltd (Lanzhou, China); the other seven rootstock varieties were obtained from Jingyan Yinong Seed Industry Technology Co., Ltd (Beijing, China) (Table S3). Seeds were surface-disinfected in 70% ethanol for 30 s and 25% bleach for 30 min, followed by three rinses with sterile water. Sterilized seeds were sown in a professional growth substrate consisting of soil and coir at a ratio of 2:1. Plants were grown under a 16 h light/8 h dark photoperiod at 25 °C/20 °C. Twenty seedlings were planted for each rootstock variety. When seedlings developed three true leaves, RT-PCR was performed to confirm that the rootstocks were free of CGMMV infection before inoculation.

Although CGMMV-positive field samples were obtained during the survey, a sequence-confirmed infectious clone was used for rootstock resistance evaluation to ensure uniform and reproducible inoculation. Field-derived inocula may contain mixed infections, variable viral titers, or degraded viral particles, which could introduce experimental variability. Therefore, the infectious clone pCB-CGMMV-lnxg (GenBank accession number KY040049) was used as a standardized inoculum source for comparing CGMMV accumulation among different rootstocks.

The pCB-CGMMV-lnxg clone was constructed and maintained by the Plant Virus Laboratory, College of Plant Protection, Shenyang Agricultural University, and stored at −80 °C. Agrobacterium tumefaciens cultures harboring the CGMMV infectious clone were incubated overnight at 28 °C, harvested the following morning, and resuspended in infiltration buffer containing 10 mM 2-(N-morpholino)ethanesulfonic acid (MES, pH 5.6), 10 mM MgCl_2_, and 150 µM acetosyringone to an OD_600_ of 1.0. After incubation at room temperature for 3 h, the suspension was infiltrated into the lower two cotyledons of rootstock plants at the three-leaf stage using a one mL needleless sterile syringe. At 14 and 21 days post-inoculation (dpi), the upper leaves were collected for quantification of viral accumulation.

### Establishment and validation of endogenous reference genes in rootstocks

Because the nine rootstock varieties belonged to three different cucurbit species, namely gourd, pumpkin, and watermelon, species- or variety-specific endogenous reference primers were established before quantifying viral accumulation. Total RNA was extracted from each rootstock variety and reverse-transcribed into cDNA. Conserved regions of the *Actin* gene were amplified using degenerate primers designed from homologous sequences of related cucurbit species (Table 1).

The watermelon *Actin* sequence deposited in NCBI (GenBank accession number KY367356.1) was used as a reference for homologous amplification in the wild watermelon rootstock. In parallel, this sequence was used as a query for BLASTN searches against the pumpkin genome database (cultivar Rifu; GenBank assembly GCA_002738365.1) and the gourd genome database (cultivar USVL1; GenBank assembly GCA_003268545.1). Homologous *Actin* genes with relatively high expression levels and low tissue-specific variation were selected for cloning.

PCR products were purified, cloned into plasmid vectors, and subjected to Sanger sequencing. The obtained sequences were assembled and analyzed using DNAMAN software. Based on these aligned sequences, RT-qPCR primers were designed using Primer Premier 5.0, with primer binding sites positioned within conserved regions to ensure stable amplification. Primer specificity was validated by conventional PCR and agarose gel electrophoresis, which produced a single amplicon of the expected size for each variety (Fig. S4). The primer sequences used for nested PCR amplification and RT-qPCR validation are listed in Table 1.

### Real-time quantitative reverse transcription-PCR (RT-qPCR)

The upper fourth leaves of each rootstock plant were collected at 14 and 21 dpi with CGMMV infectious clone for RT-qPCR analysis. Total RNA was extracted from the collected plant leaves using FreeZol Reagent (Vazyme R711; Vazyme, Nanjing, China) and subsequently reverse-transcribed into complementary DNA (cDNA) using the HiScript^®^ III 1st Strand cDNA Synthesis Kit (+gDNA wiper; Vazyme, Nanjing, China). ChamQ SYBR qPCR Master Mix (Vazyme, Nanjing, China) and StepOnePlus™ Real-Time PCR System (Applied Biosystems, Foster, USA) were used for RT-qPCR.

RT-qPCR was used to quantify accumulation of the CGMMV CP gene. Gene-specific primers targeting CGMMV CP were designed using Primer Premier 5.0, with amplicon lengths set between 80 and 150 bp. Primer specificity was confirmed by BLAST analysis against the NCBI nucleotide database and by the presence of a single peak in melting curve analysis. Amplification efficiency, determined using a standard curve generated from a 10-fold serial dilution of cDNA, ranged from 95% to 105%, with R^2^ values greater than 0.99. Primer sequences are listed in Table S2.

**Table 1:** Actin reference gene amplification primers for watermelon, gourd, and pumpkin.

**Rootstock name**	**Primer name**	**Sequence** ** (5′→3′)**	**Fragment size (bp)**
Gourd	LsActin-qF	AACTATGAACTGCCTGATGGAC	217 bp
	LsActin-qR	GTCAGCGATACCAGGGAACA	
Watermelon	ClActin-qF	CTATGAACTGCCCGATGGAC	219 bp
	ClActin-qR	GTCAGCGATACCAGGGAACA	
Pumpkin	CmActin-qF	GGGCAAGTCATCACAATCGG	180 bp
	CmActin-qR	GGTAGAACCACCACTGAGAACAA	

Each 20 µL RT-qPCR reaction contained 10 µL 2 × ChamQ SYBR qPCR Master Mix, 0.4 µL of each forward and reverse primer (10 µM), 4 µL diluted cDNA template equivalent to 50 ng total RNA, and 5.2 µL nuclease-free water. Reactions were performed in triplicate for each biological replicate under the following cycling conditions: 95 °C for 30 s; 40 cycles of 95 °C for 10 s and 60 °C for 30 s, with fluorescence acquisition at the end of each extension step; followed by melting curve analysis from 60 °C to 95° C. RT-qPCR assays were performed with three biological replicates.

### Standardization and cross-species calibration of reference gene expression levels

To eliminate the influence of the basal expression background differences of the *Actin* genes in watermelon, pumpkin, and bottle gourd on the cross-species comparison of virus accumulation, prior to calculating the relative expression level of CGMMV RNA in each rootstock, the expression level of *Actin*_[species]_ in each biological sample of each rootstock variety within this batch of data was normalized, and a species-specific calibration coefficient K was established. In this study, the wild watermelon rootstock ‘Yongzhen’ was employed as a reference. First, the basal expression level of the *Actin* gene in each species, *Actin*_[species]_=2^−Ct[Actin]^, was calculated based on the average Ct value (Ct[Actin]) of the *Actin* gene in each biological sample of each rootstock variety. Then, the calibration coefficients K for pumpkin and bottle gourd were obtained as K_[species]_ = *Actin*_[Watermelon]_/*Actin*_[species]_. Finally, the 2^−ΔΔCt^ method was used to calculate the relative accumulation of CGMMV within each species. The obtained values were then multiplied by the calibration coefficient of the corresponding rootstock variety to obtain the relative virus content that can be compared horizontally at the unified cellular level, which was used to evaluate the resistance differences of different cucurbit crops to CGMMV.

In addition, since the CGMMV resistance identification of these nine rootstock varieties was carried out in two batches, in order to quantitatively compare the virus accumulation of the six rootstock varieties in the first batch and the three rootstock varieties in the second batch together, we incorporated the wild watermelon ‘Yongzhen’ from the first batch as a calibration sample into the rootstock varieties in the second batch for the correction of relative expression level. Δ_correct_ = ΔCt_2nd_ – ΔCt_1st_, where ΔCt_1st/2nd_ represents Ct_[CGMMV]_ – Ct_[Actin]_ of ‘Yongzhen’ in the first/second batch. The corrected ΔCt of all samples in the second batch is equal to the measured ΔCt minus Δcorrect.

### Western blotting

Western blotting was performed to validate CGMMV accumulation at the protein level. Total protein was extracted from leaf samples using a Plant Protein Extraction Kit (Coolaber, Beijing, China) and quantified using a bicinchoninic acid (BCA) Protein Assay Kit (Beyotime, Shanghai, China). For each sample, 20 µg total protein was mixed with 5× sodium dodecyl sulfate-polyacrylamide gel electrophoresis (SDS-PAGE) loading buffer, denatured at 95 °C for 10 min, and separated by 12% SDS-PAGE.

Proteins were transferred electrophoretically onto a 0.2 µm polyvinylidene fluoride (PVDF) membrane (Biosharp, Shanghai, China). To verify equal protein loading and transfer efficiency, the membrane was stained with Coomassie Brilliant Blue (CBB). The membrane was then blocked with 5% (w/v) non-fat milk in TBST buffer containing Tris-buffered saline and 0.1% Tween-20 for 2 h at room temperature.

For immunodetection, the membrane was incubated overnight at 4 °C with a CGMMV monoclonal primary antibody (YouLong Biotech, Shanghai, China) diluted 1:4000 in blocking buffer. After three washes with TBST for 10 min each, the membrane was incubated with horseradish peroxidase (HRP)-conjugated anti-mouse IgG antibody (Solarbio, Beijing, China) diluted 1:8000 for 1 h at room temperature. After washing, immunoreactive signals were visualized using Omni-ECL chemiluminescent substrate solution (Epizyme, Shanghai, China) and captured using a Tanon Chemiluminescence Gel Imager (Tanon, Shanghai, China). A parallel blot probed with an anti-Actin monoclonal antibody was used as a loading control.

## Results

### Identification of viral diseases in watermelons across diverse cultivation regions

**Figure 1:**
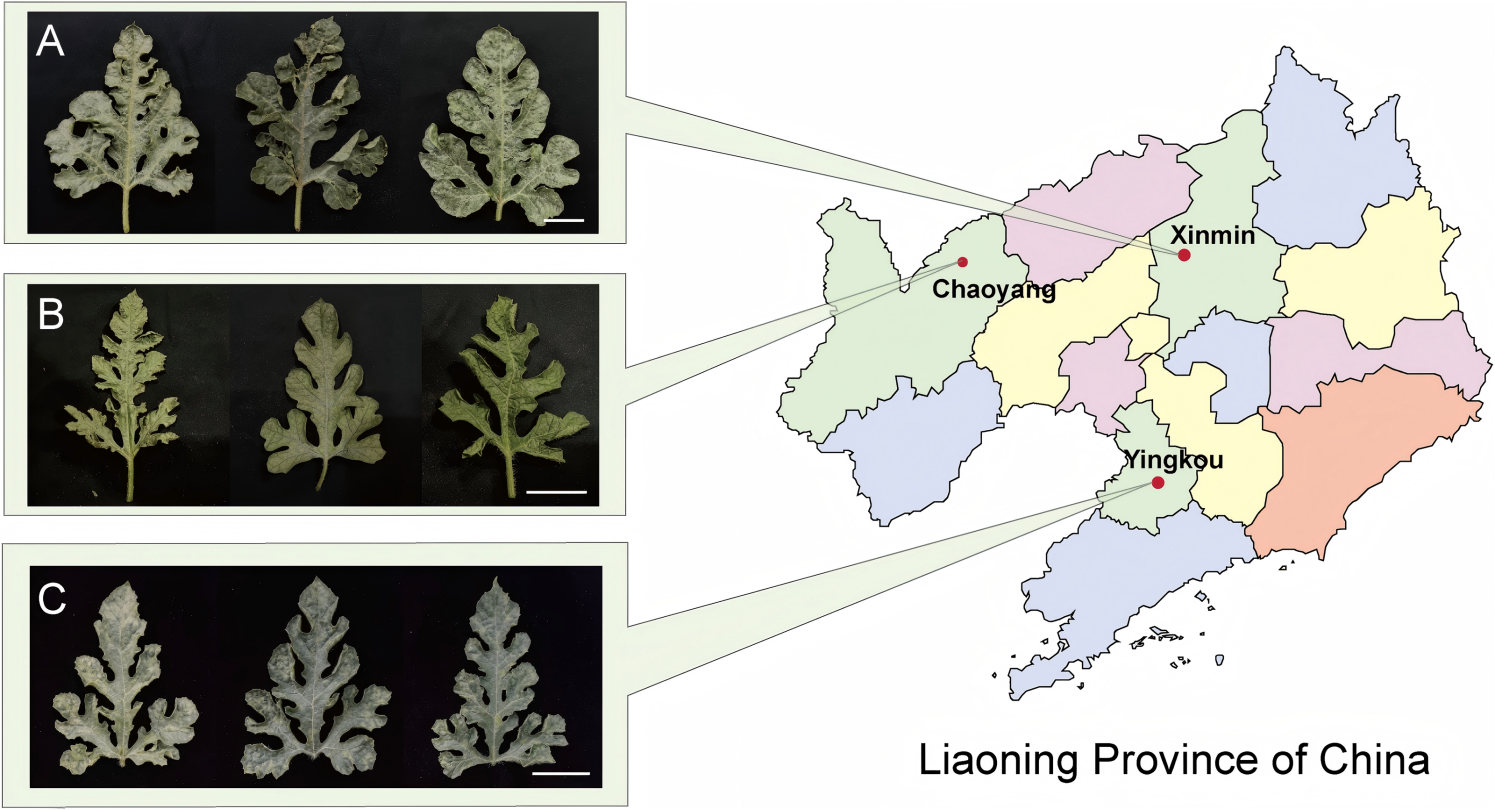
Leaf sample suspected of watermelon viral disease. (A) Xinmin City; (B) Chaoyang City; (C) Yingkou City.

**Figure 2:**
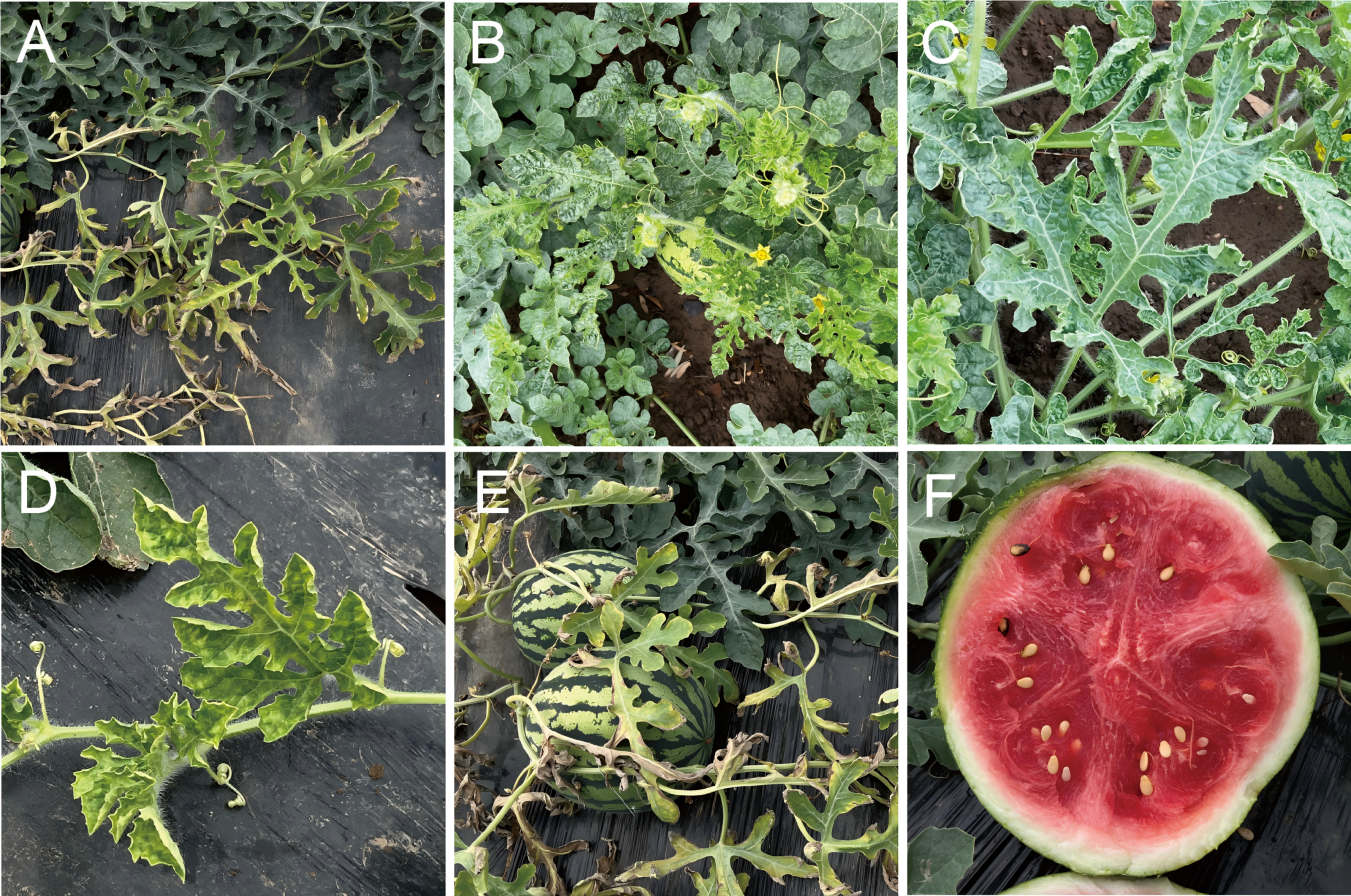
Symptoms of suspected watermelon virus disease in the field. (A–D) Mosaic and wrinkling on leaves; (E–F) rot and discoloration on fruits.

From July 2023 to August 2024, field surveys and photographic documentation were conducted in the major watermelon-producing regions of Liaoning Province, China. A total of 330 symptomatic watermelon samples were collected, specifically, 90 samples from Yingkou City, 180 from Xinmin City, and 60 from Chaoyang City (Table S1). Field investigations revealed that virus-like diseases were prevalent in both open-field and greenhouse-grown watermelon crops, with diverse symptom phenotypes observed (Fig. 1). Leaf symptoms ranged from mild to severe chlorotic mottling and wrinkling, and in severe cases, fern-like leaf deformation (Figs. 2A–2E), often accompanied by overall plant stunting. In addition, infected fruits exhibited internal pulp rot, discoloration, and a sour odor (Fig. 2F), which substantially reduced their market value.

RT-PCR screening of the 330 samples was initially performed using pooled samples (five individual samples per pool) with virus-specific primers targeting seven major watermelon-infecting viruses in China, including CGMMV, CMV, WMV, TMV, ZYMV, MYSV, and WSMoV. Among these viruses, only CGMMV and WMV were detected (Figs. S1–S3). The amplified fragments of CGMMV (654 bp) and WMV (242 bp) corresponded to the expected product sizes. The overall detection rate of CGMMV was 54.85% (181/330), whereas WMV was detected in 15.76% (52/330) of the samples. Notably, all samples collected from Shanghezhai Village (Yingkou City) were co-infected with both CGMMV and WMV. CGMMV was detected in samples from seven surveyed locations, with the exception of Yichenggong Town (Table 2; Figs. S1–S3), where only WMV was identified in samples from Chaoyang City.

**Table 2:** Results of RT-PCR identification of watermelon samples.

**City**	**Investigation site**	**No. of samples tested**	**No. of positive of watermelon virus in the tested samples**						
	**(County/town)**								
			**CGMMV**	**CMV**	**WMV**	**TMV**	**ZYMV**	**MYSV**	**WSMoV**
Yingkou	Gaizhou	45	57	0	16	0	0	0	0
	Lutun	45	24	0	0	0	0	0	0
Xinmin	Liangshan	60	31	0	0	0	0	0	0
	Lujiatun	60	28	0	0	0	0	0	0
	Yaopu	60	29	0	7	0	0	0	0
Chaoyang	Heishui	30	12	0	0	0	0	0	0
	Yichenggong	30	0	0	29	0	0	0	0

These results indicate that watermelon viral diseases are most prevalent in the Gaizhou District of Yingkou City, where CGMMV is the dominant viral pathogen, followed by WMV. Overall, CGMMV is identified as the predominant virus affecting watermelon production in Liaoning Province.

### Phylogenetic analysis of CGMMV and WMV isolates from Liaoning Province

To assess regional genetic variation, symptomatic samples were collected from three major watermelon-producing areas in Liaoning Province, namely Yingkou, Xinmin, and Chaoyang. Two isolates were obtained from each location and subjected to sequence analysis. The resulting sequences were included in phylogenetic analyses together with representative isolates retrieved from GenBank.

Sanger sequencing yielded six complete genome sequences of CGMMV-LNXG and six CP gene sequences of WMV-LNXG. Within each sampling location, the two isolates showed identical nucleotide sequences for both viruses, indicating high intra-regional homogeneity. Among different regions, slight sequence variation was observed in the CGMMV full-length genomes, whereas the WMV CP sequences were completely identical across all three locations. The complete genome sequences of CGMMV-YKXG, CGMMV-XMXG (GenBank accession number PX021799), and CGMMV-CYXG each had a length of 6,424 bp. The CP gene sequences of WMV-LNXG (GenBank accession number PV538501) were 1,087 bp in length.

**Figure 3:**
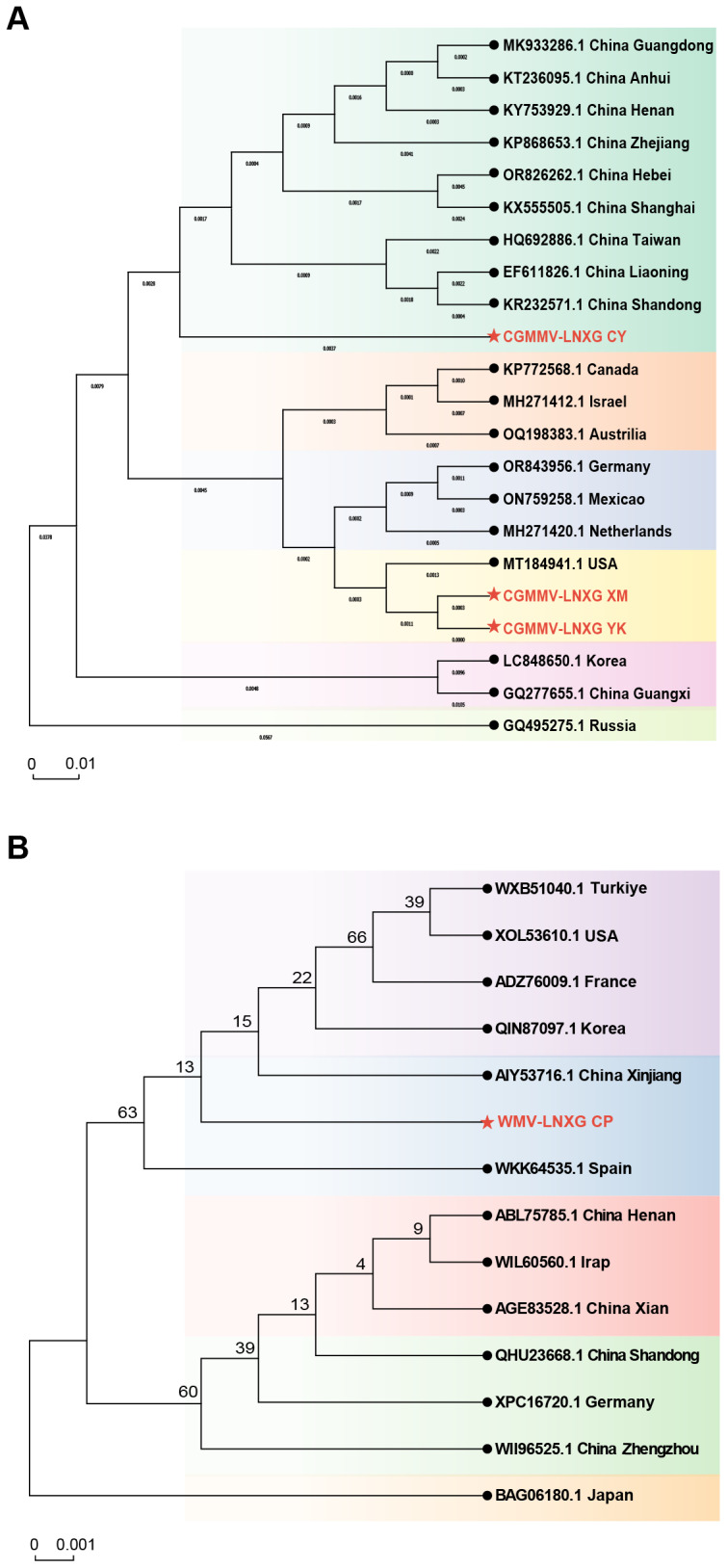
Phylogenetic tree showing the relationships among the CGMMV complete genome and WMV CP described and other Isolates from other regions at the NCBI web site. (A) A phylogenetic tree of 20 CGMMV based on complete genome with their geographic origin; (B) a phylogenetic tree of 14 WMV based on CP with their geographic origin. The evolutionary history was inferred using the Neighbor-Joining (NJ) method. The tree is drawn to scale, with branch lengths in the same units as those of the evolutionary distances used to infer the phylogeretic tree.

Phylogenetic analysis based on complete genome sequences showed that the Liaoning CGMMV isolates clustered into distinct subclades (Fig. 3A). The CGMMV-LNCY isolate grouped with previously reported Chinese isolates, including those from Guangdong, Anhui, Henan, Zhejiang, Hebei, Shanghai, Taiwan, Liaoning, and Shandong, forming a major clade. In contrast, CGMMV-LNXM and CGMMV-LNYK clustered with an isolate from the United States (MT184941), forming a separate branch, suggesting a closer genetic relationship with overseas isolates. For WMV, phylogenetic analysis based on CP sequences revealed that the Liaoning isolate (WMV-LNXG CP) clustered closely with a Xinjiang isolate (AIY53716.1), forming a well-supported clade and was more distantly related to isolates from Japan (Fig. 3B). This clade was distinct from other international isolates from Europe and North America, indicating a relatively conserved genetic background among Chinese WMV populations.

### Initial assessment of CGMMV infection across nine watermelon rootstocks

**Figure 4:**
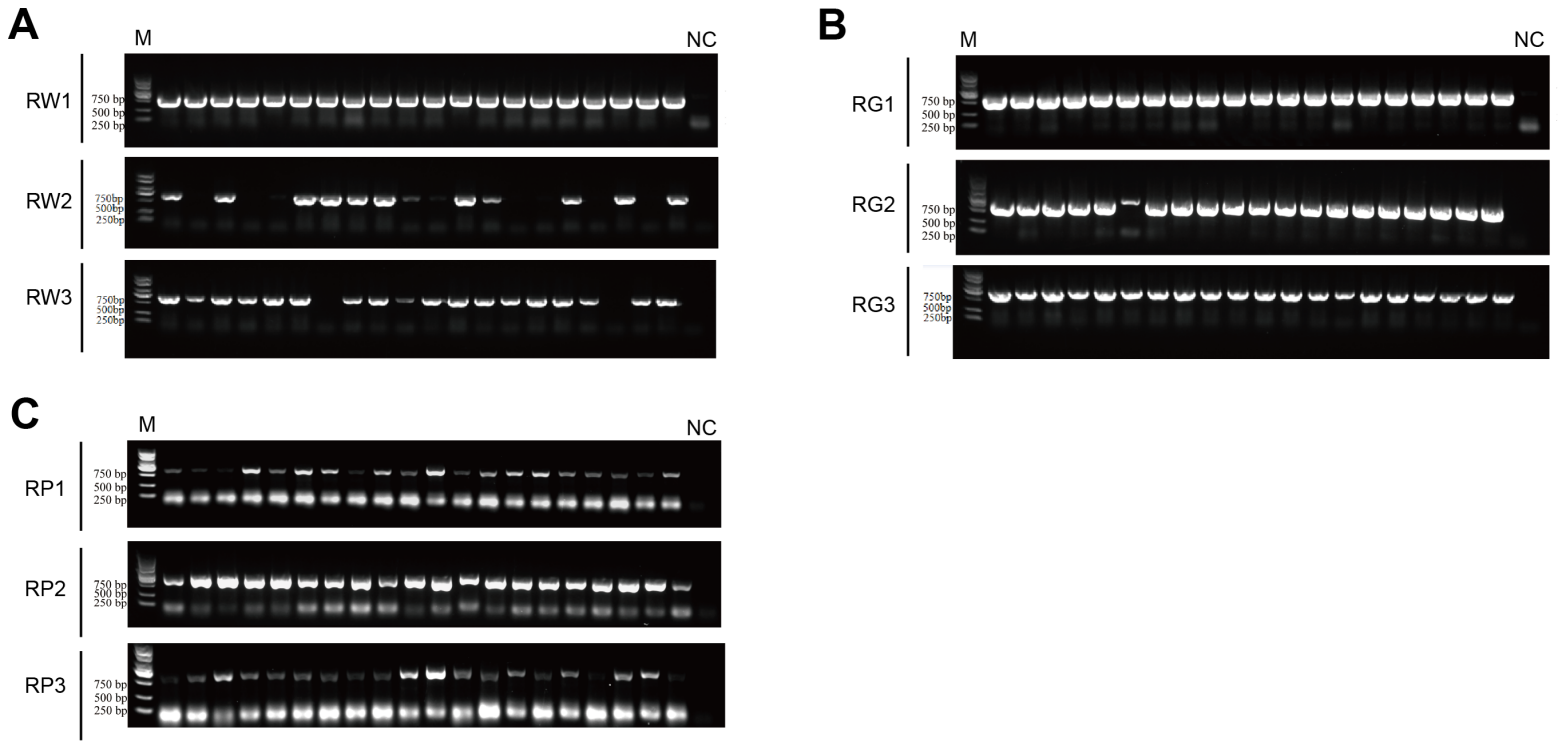
RT-PCR detection of CGMMV in nine rootstock lines at 14 dpi. Agarose gel electrophoresis of RT-PCR products amplified from (A) wild watermelon rootstocks (RW1–RW3), (B) bottle gourd rootstocks (RG1–RG3), and (C) pumpkin-type rootstocks (RP1–RP3) at 14 dpi. Each lane represents an individual plant sample. M, DNA molecular size marker; NC, negative control.

The selection of appropriate rootstocks is crucial for successful watermelon grafting and stable production. To assess the influence of rootstock variety on CGMMV infection, nine commercially available rootstocks abbreviated as RW1-3 (wild watermelon rootstock No. 1 to No. 3), RG1-3 (bottle gourd rootstock No. 1 to No. 3), and RP1-3 (pumpkin rootstock No. 1 to No. 3) were selected, with wild watermelon rootstock variety ‘Yongzhen’ serving as a control (Table S3). To ensure that all plants were initially free of the virus, seedlings at the three-true-leaf stage were tested by RT-PCR prior to inoculation, and no CGMMV infection was detected. Plants were subsequently inoculated with a CGMMV infectious clone *via Agrobacterium*-mediated delivery. Following inoculation, differences in the proportion of symptomatic plants were observed among rootstocks. RW1, RG1, RG2, RG3, and RP2 showed symptoms in all tested plants. In contrast, RW3, RP1, RP3, and RW2 exhibited lower proportions of symptomatic individuals, with RW2 showing the lowest level (Fig. 4). These results suggest that disease incidence varied among rootstocks, but incidence alone was not sufficient to determine resistance. Therefore, subsequent symptom observation, RT-qPCR, and Western blot analyses were used to evaluate the relative response of each rootstock to CGMMV.

### Comparison of the resistance to CGMMV of nine kinds of rootstocks

**Figure 5:**
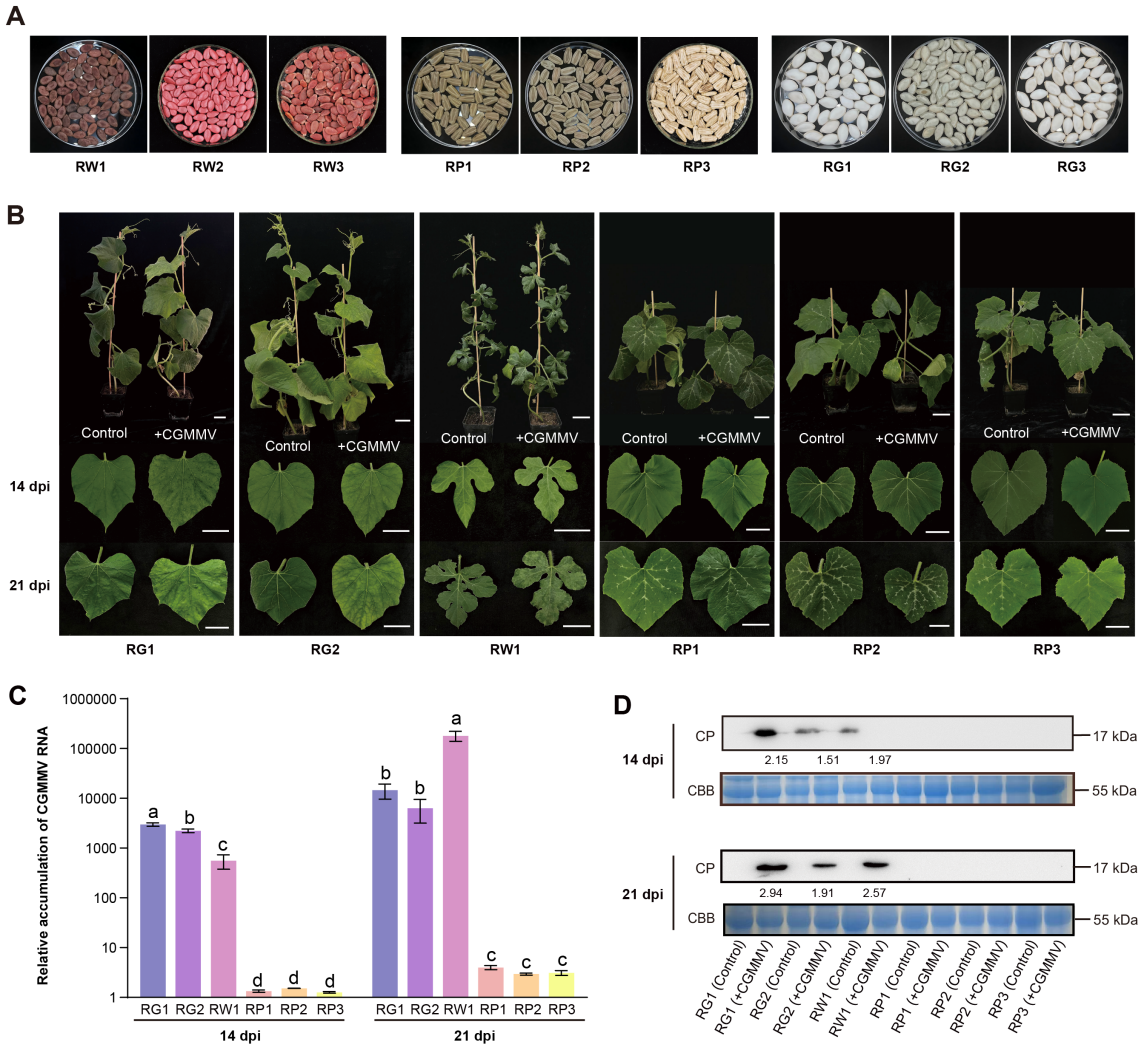
Seed morphology of different rootstocks and symptom development following CGMMV inoculation. (A) Seed morphology of nine rootstock lines (RW1–RW3, RP1–RP3, RG1–RG3). Seeds from each rootstock were shown in petri dishes. (B) Symptom development in seedlings at 14 and 21 days post-inoculation (dpi) with CGMMV. Each rootstock is shown with control (mock-inoculated) and CGMMV-inoculated plants. Upper panels show whole plants, lower panels show representative leaves. Scale bars = 5 cm. (C) Quantification of CGMMV accumulation in leaves of different rootstocks at 14 and 21 dpi. Data are presented on a log10 scale due to large variability among groups. Different letters indicate statistically significant differences (*P* < 0.05, ANOVA with Tukey’s test). (D) Western blot detection of CGMMV coat protein (CP) in leaf extracts from control and CGMMV-inoculated plants at 14 and 21 dpi. Coomassie Brilliant Blue (CBB) staining shows total protein loading. The relative intensity of CP bands is indicated below each lane.

The seeds display distinct differences in size, shape, color, and surface texture, reflecting the genetic diversity among the three types of rootstocks. Specifically, the seeds of bottle gourd rootstocks (‘Jingxin Zhensheng’, ‘Jingxin Zhenwang’ and ‘Jingxin Zhenzhuang’) are medium-sized, elongated, and light brown with slight ridges (Fig. 5A). The seeds of wild watermelon rootstock (‘Yongzhen’, ‘Zhongxu mengbao’, ‘Yezhen No. 6’) seeds are smaller, flattened, and dark brown with a rough texture (Fig. 5A). The seeds of pumpkin rootstocks (‘Jingxin Zhen No. 2’, ‘Jingxin Zhen No. 4’, ‘Jingxin Zhen No. 9’) are typically larger, oval-shaped, with a creamy-white color and smooth surface (Fig. 5A). These morphological variations are consistent with the taxonomic and genetic backgrounds of the rootstocks, which may further correlate with their differential responses to CGMMV infection.

After 14 dpi with CGMMV, mosaic mottling symptoms were observed on the leaves of ‘Jingxin Zhensheng’, ‘Jingxin Zhenwang’, and ‘Yongzhen’, whereas no visible symptoms were detected in ‘Jingxin Zhen No. 2, No. 4, and No. 9’ (Fig. 5B). Yongzhen and the three additional rootstocks (Zhongxu Mengbao, Yezhen No. 6, and Jingxin Zhenzhuang) displayed visible mosaic symptoms, whereas pumpkin rootstocks remained largely asymptomatic (Fig. S5A). At 21 dpi, the leaves of ‘Jingxin Zhensheng’, ‘Jingxin Zhenwang’, and ‘Yongzhen’ exhibited a lighter color compared to the control group, accompanied by more severe mosaic symptoms In contrast, plants of Jingxin Zhen No. 2, No. 4, and No. 9 displayed only mild wrinkling (Fig. 5B). Leaves of RG1, RG2, and RW1 displayed chlorosis and more severe mosaic symptoms compared with the control group, whereas pumpkin rootstocks showed only mild leaf wrinkling (Fig. S5A).

The accumulation levels of CGMMV genomic RNAs and CP, as determined by RT-qPCR and Western blot analysis respectively, were correlated with the severity of the observed symptoms. The wild watermelon ‘Yongzhen’ displayed the most severe mosaic mottling (Fig. 5B). Based on RT-qPCR and Western blot analyses of the first batch of six rootstock varieties, the accumulation levels of CGMMV genomic RNAs and CP were significantly higher in ‘Yongzhen’. Subsequently, two bottle gourd varieties, ‘Jingxin Zhensheng’ and ‘Jingxin Zhenwang’, also had relatively high accumulation levels. In contrast, three pumpkin rootstocks, ‘Jingxin Zhen No. 2, No. 4, and No. 9’, exhibited the strongest resistance to CGMMV (Figs. 5C, 5D). When the three rootstocks from the second batch were analyzed collectively, the same conclusion was drawn: the three tested pumpkin-type rootstocks remained the most resistant to CGMMV (Fig. S5B). The gourd-type rootstocks showed lower resistance compared to pumpkins but higher resistance than wild watermelons. Among the three wild watermelon species, ‘Yongzhen’ remained the most susceptible to CGMMV, while ‘Yezhen No. 6’ showed resistance levels equivalent to those of bottle gourd rootstocks, suggesting that genetic diversity within wild watermelon species has led to differentiation in CGMMV resistance (Fig. S5B). Pumpkin rootstocks are widely used in watermelon grafting because they often show vigorous root growth and improved tolerance to several soil-borne stresses, particularly Fusarium wilt. The results of this study demonstrate that the three tested pumpkin-type rootstocks exhibit consistently display high resistance to CGMMV, which further enhances the benefits of applying pumpkin-type rootstocks.

## Discussion

This study investigated the prevalence of viral diseases in watermelon crops in Liaoning Province , which is situated in the northeast of China, and identified CGMMV and WMV as the primary pathogens affecting local production. Among the seven viruses tested, the overall incidence of viral infection was relatively low. Field observations were consistent with laboratory results, with only a limited number of plants exhibiting typical virus-like symptoms. In addition, several viruses commonly reported in tropical and subtropical regions were not detected. The low prevalence of viral infections in Liaoning may be attributed to its distinct ecological and agricultural conditions. Specifically, (i) the temperate continental monsoon climate, characterized by cold and dry winters, is unfavorable for the overwintering of insect vectors (aphids and whiteflies) and the viruses they transmit (Jeger et al., 2023; Van Munster, 2020); (ii) the relatively short growing season may limit successive cycles of virus replication and transmission (Krenz, Niehl & Krczal, 2024); and (iii) standardized large-scale agricultural practices, including crop rotation, field sanitation, and uniform management, may reduce the availability of primary inoculum sources (Al-Musawi, Vona & Kulmány, 2025).

Although only CGMMV and WMV were detected among the seven commonly reported watermelon-infecting viruses, the absence of other viruses in the present survey does not definitively exclude their occurrence in Liaoning Province. Viruses such as Papaya ringspot virus (PRSV), poleroviruses, begomoviruses (geminiviruses), and other emerging cucurbit-infecting viruses may still be present but remained undetected under the current experimental conditions. Nevertheless, given that these viruses have not been previously reported in watermelon production systems in this region, their incidence is likely to be low. In addition, virus-like symptoms observed in the field may arise from mixed infections, viral titers below the detection threshold, or abiotic stress factors rather than single-virus infections. Therefore, despite the relatively low diversity of detected viruses, further investigations employing high-throughput sequencing technologies are warranted to achieve a comprehensive characterization of the watermelon virome in Liaoning Province and to uncover potential cryptic or low-abundance viral agents.

CGMMV spreads efficiently through mechanical transmission and contaminated seeds, facilitating its global dissemination. Phylogenetic analyses of CGMMV and WMV isolates from Liaoning reveal distinct evolutionary patterns shaped by geography and epidemiology. CGMMV-LNCY clusters with previously reported Chinese isolates, indicating a relatively conserved domestic lineage, while CGMMV-LNXM and CGMMV-LNYK are closely related to a US isolate (MT184941), suggesting potential international introduction. Substantial seed trade between Liaoning and California from 2015-2020, with 2.1% CGMMV contamination in commercial batches, highlights seed-mediated long-distance dissemination and the virus’s potential for transboundary spread. For WMV, the Liaoning isolate (WMV-LNXG CP) exhibited high genetic similarity to a Xinjiang strain, suggesting either a shared origin or convergent evolutionary pressures rather than direct introduction. Given Xinjiang’s proximity to Central Asia and its favorable conditions for aphid populations, both trade and vector-mediated transmission may contribute to WMV spread (Desbiez & Lecoq, 2004).

Grafting is widely recognized as an effective strategy to enhance plant resistance, mitigate continuous cropping constraints, and control soil-borne diseases such as Fusarium wilt in watermelon production (Abu-Nasser & Abu-Naser, 2018). With the advancement of cultivation techniques and increasing economic returns—particularly through industrialized seedling production—the adoption of grafting in watermelon cultivation has expanded and is expected to become a dominant production system. Currently, rootstocks used in watermelon grafting are primarily classified into three categories: pumpkin, bottle gourd, and wild watermelon rootstocks. Following infection, plant viruses typically move through the phloem sieve elements to the root system, where they may replicate before systemic dissemination *via* vascular tissues (Heinlein, 2015). As the origin of the belowground system in grafted plants, rootstocks may play a critical role in determining whether viruses can establish and proliferate in root tissues. In this study, nine commercially available rootstocks belonging to three types of rootstocks were evaluated for resistance to CGMMV.

Agrobacterium-mediated delivery of CGMMV infectious clones has been widely used in cucurbit hosts, providing a stable and reproducible system for pathogenicity and viral accumulation assays. Previous studies have shown host-dependent variation in viral accumulation following agroinfiltration, supporting its reliability for comparative resistance evaluation (Zheng et al., 2015; Nayaka et al., 2023; Liu et al., 2025). Quantitative detection results after virus inoculation demonstrated that among the tested rootstock varieties, pumpkin-type rootstocks exhibited significantly stronger resistance to CGMMV compared to gourd-type rootstocks and wild watermelon subspecies. Most pumpkin-type rootstocks have undergone selective breeding *via* long-term hybridization and targeted screening, with continuous purification of disease-resistant genotypes in breeding programs, resulting in homogenized genetic backgrounds and uniform resistance. Compared to pumpkin-type rootstocks, bottle gourd-type rootstocks lack systematic disease resistance improvement, with local germplasm randomly preserving individual plants with varying resistance levels, resulting in significant differences among varieties. Wild watermelons possess complex origins, encompassing African wild native species, semi-domesticated local resources, and wild relatives of cultivated watermelons. Geographic isolation in native habitats leads to population genetic differentiation, resulting in the natural coexistence of both disease-resistant and susceptible genes without uniform disease resistance screening. This explains the reason for the significant intraspecific resistance variation observed among the three wild watermelon rootstocks in this study. In China, the ‘Jingxin Zhen’ series of pumpkin rootstocks have been developed and applied in production for their favorable grafting compatibility and improved disease resistance relative to ungrafted plants (Meng et al., 2023). Specifically, ‘Jingxin Zhen No. 4’ has been documented as an effective watermelon rootstock that enhances growth and reduces Fusarium wilt severity. Our results further demonstrate the ‘Jingxin Zhen’ series of pumpkin rootstocks strong resistance to CGMMV.

Soluble sugar content is a key determinant of watermelon fruit quality and strongly influences consumer preference and market value. Previous studies have shown that pumpkin rootstocks may reduce soluble sugar content in grafted watermelon fruits, whereas watermelon rootstocks (*e.g.*, ‘Wild Watermelon No. 2’) promote sugar accumulation in the fruit flesh (Yetisir & Sari, 2003; López-Galarza et al., 2004; Kyriacou et al., 2017). As a wild watermelon species belonging to the same genus as cultivated watermelons, it exhibits stronger graft compatibility compared to other non-watermelon rootstocks and offers significant advantages in preserving the sweetness and flavor of watermelon fruits. The three wild watermelon rootstocks in this study showed significant intraspecific resistance variation, among which ‘Yezhen No. 6’ exhibited strong resistance to CGMMV and could serve as an excellent candidate rootstock combining CGMMV resistance with high watermelon quality. Meanwhile, it is necessary to explore more wild watermelon resources that combine strong disease resistance with desirable fruit quality traits for development into rootstocks.

## Conclusions

In summary, the most damaging viruses currently affecting major watermelon-producing regions in Liaoning Province are CGMMV and WMV. The CGMMV-LNXG isolate is phylogenetically closest to the isolate from the USA, while WMV-LNXG isolate shows the closest genetic relationship with isolate from Xinjiang. Empirical evidence indicates that among the nine rootstock genotypes evaluated, pumpkin-derived rootstocks conferred the highest and most consistent resistance to CGMMV, exhibiting exceptional stability across diverse cultivars. In contrast, bottle gourd rootstocks demonstrated moderate resistance to CGMMV but were markedly less effective than pumpkin-type rootstocks in both absolute resistance level and intra-genotypic uniformity. Wild watermelon accessions, characterized by substantial genetic diversity, displayed pronounced intersubspecific variation in CGMMV resistance. Collectively, this study outlines genus- and species-level patterns of resistance differentiation among cucurbit rootstocks, thereby informing evidence-based rootstock selection frameworks that jointly optimize disease resistance and key agronomic traits. Furthermore, the identified resistant germplasm and associated resistance profiles provide essential resources and conceptual foundations for molecular marker-assisted breeding and mechanistic investigations into CGMMV resistance in *Cucurbitaceae*.

##  Supplemental Information

10.7717/peerj.21603/supp-1 Supplemental Information 1 Actin Cloning of the Three Rootstock Species

10.7717/peerj.21603/supp-2 Supplemental Information 2 Original gel image of RT-PCR detection for CGMMV in Jingxin Zhensheng at 14 dpi

10.7717/peerj.21603/supp-3 Supplemental Information 3 Original gel image of RT-PCR detection for CGMMV in Jingxin Zhenwang at 14 dpi

10.7717/peerj.21603/supp-4 Supplemental Information 4 Original gel image of RT-PCR detection for CGMMV in Yongzhen at 14 dpi

10.7717/peerj.21603/supp-5 Supplemental Information 5 Original gel image of RT-PCR detection for CGMMV in Jingxin Zhen No.2 at 14 dpi

10.7717/peerj.21603/supp-6 Supplemental Information 6 Original gel image of RT-PCR detection for CGMMV in Jingxin Zhen No.4 at 14 dpi

10.7717/peerj.21603/supp-7 Supplemental Information 7 Original gel image of RT-PCR detection for CGMMV in Jingxin Zhen No.9 at 14 dpi

10.7717/peerj.21603/supp-8 Supplemental Information 8 Original gel image of RT-PCR detection for CGMMV in Zhongxu Mengbao and Yezhen No. 6 at 14 dpi

10.7717/peerj.21603/supp-9 Supplemental Information 9 Raw data of viral accumulation in rootstocks (Yongzhen, Jingxin Zhensheng, Jingxin Zhenwang) infected with CGMMV at 14 and 21 days post-inoculation detected by RT-qPCR

10.7717/peerj.21603/supp-10 Supplemental Information 10 Raw data of viral accumulation in rootstocks (Yongzhen,Jingxin Zhen No. 2, No. 4, and No. 9) infected with CGMMV at 14 and 21 days post-inoculation detected by RT-qPCR

10.7717/peerj.21603/supp-11 Supplemental Information 11 Raw data of viral accumulation in rootstocks (Yongzhen, Zhongxu Mengbao, Yezhen No. 6, and Jingxin Zhenzhuang) infected with CGMMV at 14 days post-inoculation detected by RT-qPCR

10.7717/peerj.21603/supp-12 Supplemental Information 12 Raw data of viral accumulation in rootstocks (Yongzhen, Zhongxu Mengbao, Yezhen No. 6, and Jingxin Zhenzhuang) infected with CGMMV at 21 days post-inoculation detected by RT-qPCR

10.7717/peerj.21603/supp-13 Supplemental Information 13 MIQE Checklist

10.7717/peerj.21603/supp-14 Supplemental Information 14 Original Western blot result image of rootstocks at 14 days post CGMMV inoculation

10.7717/peerj.21603/supp-15 Supplemental Information 15 Original CBB-stained gel image at 14 days post inoculation (dpi) with CGMMV

10.7717/peerj.21603/supp-16 Supplemental Information 16 Original Western blot result image of rootstocks at 21 days post CGMMV inoculation

10.7717/peerj.21603/supp-17 Supplemental Information 17 Original CBB-stained gel image at 21 days post inoculation (dpi) with CGMMV

10.7717/peerj.21603/supp-18 Supplemental Information 18 Watermelon survey areas in Liaoning Province

10.7717/peerj.21603/supp-19 Supplemental Information 19 Classification and characteristics of six types of rootstocks

10.7717/peerj.21603/supp-20 Supplemental Information 20 Primers used in this study

10.7717/peerj.21603/supp-21 Supplemental Information 21 The infiltration success rate of CGMMV after inoculation on different rootstocks

10.7717/peerj.21603/supp-22 Supplemental Information 22 Supplemental figures

## Additional Information and Declarations

### Data Availability

The following information was supplied regarding data availability: The raw measurements are available in the Supplemental Files. The raw sequence data are available at NCBI: PX021799, PV538501.
