# Supplementary material for: Molecular detection of watermelon virus disease in Liaoning Province, China, and resistance evaluation of nine rootstocks to cucumber green mottle mosaic virus
Source: PeerJ. 2026 Aug 10;14:e21603. doi: 10.7717/peerj.21603 (PMC13471747; doi:10.7717/peerj.21603)
Supplement: Supplementary file 1 [file peerj-14-21603-s001.docx]

Experimental Protocol: Cloning of Actin Genes from Watermelon, Pumpkin and Bottle Gourd

**[Bottle Gourd]**

The Actin gene sequence of watermelon was used for BLAST search against the bottle gourd genome. Three Actin genes were obtained as shown below, and the one with the longest alignment length was selected for subsequent cloning.

Query=Length=1134ScoreE

Sequencesproducingsignificantalignments:(Bits)Value

Lsi10G0070901198900

Lsi01G0113001123600

Lsi03G0081001109400

**Legend:** five_prime_UTRCDSthree_prime_UTR

TTTCCTTCTTCCATTCCTTCCTTCTCTCCATTTTCGATCTCACCTTCTCGCCTTTGCAACCGAGAGAGACTGAGCCTGAAAAAGGAGAGTAGGCCGTCGATTCCCTCACTTGGCTCTTCCATTTTCCTTCTCTTCTTCGTTTTCTTCACTTCCTAAGGTATGATTTCCTTTCGATCTGACTTTCTTTAGATCTCTTATTCTGCTTTTCATTCAGATTTCGATTTGCTTTTGTTGCCATTTCTGTTTTTGTGTTTATCACTCTTATGAGCTTCGATCTGGTTGTTTTCTTTCTTTCTTTTTTTTTTTTTTTTTGTTTTTTTTTTTTTTTTTTTTTCTTTCTTTTTTTTTTTTTTTTTGTTTTTTTTTTTTTTTTTTTTTCTTTCTTTTTTTTTTTTTTTTTGTTTTTTTTTTTTTTTTTTTTTCTTTCTTTTTTTTTTTTTTTTTGTTTTTTTTTTTTTTTTTTTTTCTTTCTTTTTTTTTTTTTTTTTGTTTTTTTTTTTTTTTTTTTTTCTTTCTTTTTTTTTTTTTTTTTGTTTTTTTTTTTTTTTTTTTTTCTTTCTTTTTTTTTTTTTTTTTGTTTTTTTTTTTTTTTTTTTTTCTTTCTTTTTTTTTTTTTTTTTGTTTTTTTTTTTTTTTTTTTTTCTTTCTTTTTTTTTTTTTTTTTGTTTTTTTTTTTTTTTTTTTTTCTTTCTTTTTTTTTTTTTTTTTGTTTTTTTTTTTTTTTTTTTTTATCAATCTCTGCTTTGGTTTTTGTTGATCATCAGATTTTGTTTTAGCTTCTAGCTCTTCGATTCTCTTTTTAAACAGTGGTTGTTTTAGTTCTGAGTTTTGTAATATGAATCATGTGATTTGTAAATCGTAATGCTGTTTACTGCTGTAAGGGATACTTTTTCTTTACTTTCTTTTTATCTGTTCTGGAATCTGTTTGGTTTGTGAAGACTCGGTGACATTTCGATCGGCATTTGCAATCAGATTCTGAAATTTCAGTGGCTGATCTTTGACCTCGTGCTTGTTGTTATTGAAATTTTGCGATACTGGAGCTGAGATCTTGTATTTCTCGGAAATCGCTTGTAATTTTCTCGATGCAATTGAGATTCTTATAGCATCTTTTCCACTGCCATATTCGTCTGTGGTCTGCATGACTGGACTTATGAAGAAATTTAATTAAAGATCTCAGCTACTTGCATTGTTCTGTCGATACTGATATTGAAATTTGATTGACTCAGGATCGATTATTTGCTGACTTTACTAATAGGATTCTTAGATTAAAATCGTGTTGCTTACAGTTCTCCCTAATGCGTCTGTTTCGCCTCTTGTTTCCCTGTATTTGTTTTAACGGAATATATGTTCTAGATTTCACCTGTCACCTATGACAAGAATCAATAGTTTCTTTTGAATCCGATCTATTTCTTCCTGTAAAATTGCAGTATTTTTAGGTGTTTCCCATTTATATGATTGTTTAGACACAAGTAGAAGCAAGATGGGGTTTTGCAGTTGAAGTATTATTAGGTGGCGATGTTGTGATTTCTACTCTTGGCCTTGTATTACAAAATGGATTGTCTGTTTTATGCTTACAGCTTACTGCCAATTCAACAATGCATTGCACAGATGTGCTTCGTATTCTGGGTTATAATTTGCTCATTTGTGTGTATTTTTCAGTATCTATAAAAGATGGCTGATGCTGAGGATATCCAGCCACTTGTTTGCGACAATGGAACCGGAATGGTGAAGGTACAATCACTGACTTTCAAACATATAGCCGTTTTTTTTGTTGTAGATCTACTTACGTTCTCAATAACTATAACTGTTTGTATGGGTATGTTTTCTTTTGCCTTGAGACTGGTAATAGGTTTGAAGTAATCAAATTTTAATTTGTAGGCTGGATTTGCCGGCGATGATGCTCCCAGGGCAGTTTTTCCCAGTATTGTTGGTCGACCTCGACACACTGGTGTCATGGTGGGTATGGGCCAGAAAGATGCTTACGTTGGTGACGAGGCCCAATCCAAAAGAGGTATTCTTACCTTGAAATATCCCATTGAGCATGGTATTGTCAGCAACTGGGATGATATGGAAAAGATTTGGCATCACACGTTCTACAATGAGCTTCGTGTTGCACCTGAAGAACACCCAGTGCTTCTTACTGAAGCTCCTCTCAATCCTAAAGCCAACAGAGAAAAGATGACACAAATCATGTTTGAAACTTTTAATGTACCTGCCATGTATGTTGCCATCCAGGCCGTTCTATCTCTGTACGCCAGTGGTCGTACAACAGGTTCACATCCATTTCCTGCTTTCATCTTAAATTTGGGACTCTTAGCCTTTGATTTGGATAAAAATTTAATGAATTGTTTGAATAGGTATCGTGCTCGACTCTGGTGATGGTGTTAGTCACACCGTGCCAATCTATGAGGGTTATGCTCTACCCCATGCTATCCTCCGTCTTGACCTTGCTGGTCGTGATCTAACTGATGCTTTAATGAAGATTCTCACTGAGAGAGGGTACATGTTCACAACCACTGCCGAACGGGAAATTGTCCGTGACATGAAAGAGAAGCTTGCTTATGTTGCACTCGACTATGAGCAGGAACTCGAGACTGCTAAGAGTAGCTCCTCAATTGAGAAGAACTATGAACTGCCTGATGGACAAGTCATCACAATTGGAGCTGAGAGATTCCGTTGCCCCGAAGTTCTTTTCCAGCCATCCCTCATCGGAATGGAAGCTGCTGGAATCCACGAGACTACCTACAACTCAATTATGAAGTGTGATGTGGATATCAGAAAAGATTTGTATGGCAACATTGTTCTCAGTGGTGGTTCTACCATGTTCCCTGGTATCGCTGACCGTATGAGCAAAGAGATCACAGCTCTTGCTCCCAGCAGCATGAAAATCAAGGTCGTTGCACCACCAGAAAGGAAATACAGTGTCTGGATTGGAGGGTCAATCCTTGCATCGCTCAGCACCTTCCAGCAGGTGAGATACTCCCTACATTCACTCTTAACCTTTCTTTTGTTACCTTATCTGGCACTAATACTTGGTGTGTTTTACCAGATGTGGATTTCAAAGGGCGAGTACGACGAATCTGGTCCATCCATTGTCCACAGGAAGTGCTTCTAAGTTCAATATGTGCTTTGAATGGTGAGTTATTTCCTTTAGTTGGCTTTTTTTGTGTCATGTGTAATGTGAACTCTTTCGGTTGAGCACGGATACGGGTCACGGAAGGAGGGTATATTTCTGAGGTCATTCATTCGTATGCCCATTGCTATCCCCCTACCTCTATGAGTTTGAGATTCGTAATACGCAGTATTGACGGGTTAAGAAGTAATTGACCCTCTGGATGTTTATGTTGTTGGTGGGCAAAAAACAACTTTTCTTCCAAAATCTTTCCCTGCCTGCCTTTGCCAAATCTGTGATGCAACCATGTCTTTTTTTTAGTAGGAGATGATTGTGATGTTTTTTCCCCCCTTTATCCTTCTCCCTTTTTCTCTCCTATTTCCTATTCCAAGTTCTTTCTTTTTTCGAGAACATTAATGTTAATAGTTATTGTAAGACAAATATTAGTCAGTTTGCTCCTTGCCGTCATAAACTTGCTATGCAAAATGATATTTTATTATTGGCTTTGAAGCTTTTTATAAGGGCATCATCATATTCATATTTATATTACTTCTTTCCATCCTCTTAATTCTCTATTAATATTCATTTCTCCAATTTTTGCTTCTTAATTCCCCCCCATCACTTTCACAAAGCTAGATTAGATTGGAGCCTGTGGGACCCCAACAATTTAGTATAGGTGAAGTTAAATTCTGATATTTTTGTTTAAATGTCCATAAAATTGAGAAATATTGAAAATATATTAGAGGTTGTTATTGAT

**CDS sequence:**

ATGGCTGATGCTGAGGATATCCAGCCACTTGTTTGCGACAATGGAACCGGAATGGTGAAGGCTGGATTTGCCGGCGATGATGCTCCCAGGGCAGTTTTTCCCAGTATTGTTGGTCGACCTCGACACACTGGTGTCATGGTGGGTATGGGCCAGAAAGATGCTTACGTTGGTGACGAGGCCCAATCCAAAAGAGGTATTCTTACCTTGAAATATCCCATTGAGCATGGTATTGTCAGCAACTGGGATGATATGGAAAAGATTTGGCATCACACGTTCTACAATGAGCTTCGTGTTGCACCTGAAGAACACCCAGTGCTTCTTACTGAAGCTCCTCTCAATCCTAAAGCCAACAGAGAAAAGATGACACAAATCATGTTTGAAACTTTTAATGTACCTGCCATGTATGTTGCCATCCAGGCCGTTCTATCTCTGTACGCCAGTGGTCGTACAACAGGTATCGTGCTCGACTCTGGTGATGGTGTTAGTCACACCGTGCCAATCTATGAGGGTTATGCTCTACCCCATGCTATCCTCCGTCTTGACCTTGCTGGTCGTGATCTAACTGATGCTTTAATGAAGATTCTCACTGAGAGAGGGTACATGTTCACAACCACTGCCGAACGGGAAATTGTCCGTGACATGAAAGAGAAGCTTGCTTATGTTGCACTCGACTATGAGCAGGAACTCGAGACTGCTAAGAGTAGCTCCTCAATTGAGAAGAACTATGAACTGCCTGATGGACAAGTCATCACAATTGGAGCTGAGAGATTCCGTTGCCCCGAAGTTCTTTTCCAGCCATCCCTCATCGGAATGGAAGCTGCTGGAATCCACGAGACTACCTACAACTCAATTATGAAGTGTGATGTGGATATCAGAAAAGATTTGTATGGCAACATTGTTCTCAGTGGTGGTTCTACCATGTTCCCTGGTATCGCTGACCGTATGAGCAAAGAGATCACAGCTCTTGCTCCCAGCAGCATGAAAATCAAGGTCGTTGCACCACCAGAAAGGAAATACAGTGTCTGGATTGGAGGGTCAATCCTTGCATCGCTCAGCACCTTCCAGCAGATGTGGATTTCAAAGGGCGAGTACGACGAATCTGGTCCATCCATTGTCCACAGGAAGTGCTTCTAA

Expression patterns of three Actin genes in bottle gourd: their expression levels were relatively low and stable in leaves, while higher in flowers, fruits and stems.

LsActin-F0：TGTGCTTCGTATTCTGGGTT

ClActin-R0：ATTCGTCGTACTCGCCCTTT(rc：AAAGGGCGAGTACGACGAAT)

LsActin-F0+ClActin-R0=1471 bp

**[Watermelon]**

CATTCCTTCCTTCTCTCCATTTCGATCTCACCTTCTCGCCTTTGCAACCGAGAGAGACTGAGCCTGAAAAAGGAGAGTAGGCCGTCGATTCCCTCACTTGGCTCTTCCATTATCCTTCTCTTCTTCGTTTTCTTCACTTCCTAAGGTACGGTTTCCTCTCGATCTGGTTTTCTTTAGATCTCTTATTCTGCTTTTCATTCAGATTTCGATTTGCTTTTCTTGCCATTTCTTGTTTTTTTTTTTCTCTTTATCACTCTTATGAGCTTCGATCTGCAATCTCTGCTTTGGTTTTCGTTGATCATCAGATTTTTCTTTACCTTCTAGCTCTTCGATTCTCTTTTTAAACAGTGGCTGTGTTTAGTCCTGAGTTTTGTATAATGTGAATCATGTGATTTGCAAATCGTAATGCTGTTTACTACTGTAAGGGATACTTTTTCTTTACTTTCTTTTTATCTGTTCTGGAATCTGTTTGGTTTGTGAGGACTCGGTAACATTTCGATCGACATTTGCAATCAGATTCTGAAATTTCAGTGGCTGATCTTTGACCTTCTGCTTGTTGTTATTGAAATTTTGCTATACTGGAGCTGAGATCTTGTATTTTTCGGAAATCGATTGTAGTTTTCTAGATGCAATTCGGATTTCTATAGCAACTTGTTTACTCCCATATTTCCCTATGGTCTGCATGACTGGACTTGTGAAGAAATTCAATTAAAGGTCTCAGCTGCATTGTTCTGTTGATAATGATGTTGAAATTTGATGGACTGAGGATGGGTTATTTTCCGATGTAACTAATAGGACTCTTAGACTATAAATCGTGTTGTATTACAGTTCTCCCTTCTGCATCTGCTTGGCCTCTTGTTTCCCTGTATTGTTTTTAATGGTTGTATGTTCTAGATTTCGCCCATCACCTTTGCCAAGAATCAATATTTTCTTTAAAGCCAATCCATTTCTTCCTGTAGAATTCCAGTCTTTTTAGCCGTTTCCTGCTTATTTGATTGTTTGGATACGAGTAGAAGCAAGATGGGTTTTTGCAGTAGAAGTATTATAGGTGGCAGATGTTTATGATTTCTACTCTGGGCCTTGTATTACAAAGTGGATTGTCTGTTTTATGTTACACCTTACTGCCAATTCAACAATGCATTGCACAGATGTGCTTCATATTTTATGTTACAATTTGCTCATTCGTGTGTATTTTTCAGTATCTATAAAAGATGGCTGATGCTGAGGATATCCAGCCACTTGTTTGTGACAATGGAACTGGAATGGTGAAGGTACAATCACTGACCTTAAAACATGAAGCTGTTTTTCTTGTTATTGACCTATTTACGTGCTCAATAATTATAACTGTTTTTATGGGTATGTTTTCTTTTGCCTTGAGACTGGTAATAAGTTTGAAGTAATCAAATTTTAATATGTAGGCTGGATTTGCCGGTGATGATGCTCCCAGAGCAGTGTTTCCCAGTATTGTTGGTCGACCTCGACACACTGGTGTCATGGTGGGTATGGGCCAGAAAGATGCTTACGTTGGTGACGAGGCCCAATCCAAAAGAGGTATTCTTACCTTGAAATATCCCATTGAGCATGGTATTGTCAGCAACTGGGATGATATGGAAAAGATTTGGCATCACACATTCTACAATGAGCTTCGTGTTGCACCTGAAGAACACCCAGTGCTTCTTACCGAAGCTCCTCTCAATCCTAAAGCCAACAGAGAAAAGATGACACAAATCATGTTTGAAACTTTCAATGTACCTGCCATGTATGTTGCCATCCAGGCCGTTCTATCTCTGTATGCCAGTGGTCGTACAACAGGTTCACATCCATTTCCTGCTTCCATCTTAAATTTGGGACGAAGCCTTTGATTTGGATAAATATTTAATGAATTGTTTGAATAGGTATTGTGCTCGACTCTGGTGATGGTGTTAGTCACACTGTGCCAATCTATGAGGGTTATGCTCTACCCCATGCTATCCTCCGTCTGGACCTTGCTGGTCGTGATCTAACTGATGCTTTGATGAAGATTCTCACTGAGAGAGGGTACATGTTCACAACCACTGCCGAACGGGAAATTGTCCGTGACATGAAAGAGAAGCTTGCTTATGTTGCACTCGACTATGAGCAGGAACTGGAGACTGCTAAGAGTAGCTCCTCAATTGAGAAGAACTATGAACTGCCCGATGGACAAGTCATCACAATTGGAGCTGAGAGATTCCGTTGCCCCGAAGTTCTTTTCCAGCCATCCCTCATTGGAATGGAAGCTGCTGGAATCCACGAGACTACCTACAACTCAATTATGAAGTGTGATGTGGATATCAGAAAAGATTTGTATGGAAACATTGTTCTCAGTGGTGGTTCTACCATGTTCCCTGGTATCGCTGACCGTATGAGCAAAGAGATCACAGCTCTTGCTCCCAGCAGCATGAAAATCAAGGTCGTTGCACCACCAGAAAGGAAATACAGTGTCTGGATTGGAGGATCAATCCTTGCATCGCTCAGCACCTTCCAGCAGGTGAGATATTCCCTACATTCACGCTTAACCTCTCTTTTGTTCCCATGTCCGGCACTAACTAATACTTGTGTTTTACCAGATGTGGATTTCAAAGGGCGAGTACGACGAATCTGGCCCATCCATTGTCCACAGGAAGTGCTTCTAAGTTCAATATGTGCTTTGAATGGTGAGTTAT

**CDS sequence**

ATGGCTGATGCTGAGGATATCCAGCCACTTGTTTGTGACAATGGAACTGGAATGGTGAAGGCTGGATTTGCCGGTGATGATGCTCCCAGAGCAGTGTTTCCCAGTATTGTTGGTCGACCTCGACACACTGGTGTCATGGTGGGTATGGGCCAGAAAGATGCTTACGTTGGTGACGAGGCCCAATCCAAAAGAGGTATTCTTACCTTGAAATATCCCATTGAGCATGGTATTGTCAGCAACTGGGATGATATGGAAAAGATTTGGCATCACACATTCTACAATGAGCTTCGTGTTGCACCTGAAGAACACCCAGTGCTTCTTACCGAAGCTCCTCTCAATCCTAAAGCCAACAGAGAAAAGATGACACAAATCATGTTTGAAACTTTCAATGTACCTGCCATGTATGTTGCCATCCAGGCCGTTCTATCTCTGTATGCCAGTGGTCGTACAACAGGTATTGTGCTCGACTCTGGTGATGGTGTTAGTCACACTGTGCCAATCTATGAGGGTTATGCTCTACCCCATGCTATCCTCCGTCTGGACCTTGCTGGTCGTGATCTAACTGATGCTTTGATGAAGATTCTCACTGAGAGAGGGTACATGTTCACAACCACTGCCGAACGGGAAATTGTCCGTGACATGAAAGAGAAGCTTGCTTATGTTGCACTCGACTATGAGCAGGAACTGGAGACTGCTAAGAGTAGCTCCTCAATTGAGAAGAACTATGAACTGCCCGATGGACAAGTCATCACAATTGGAGCTGAGAGATTCCGTTGCCCCGAAGTTCTTTTCCAGCCATCCCTCATTGGAATGGAAGCTGCTGGAATCCACGAGACTACCTACAACTCAATTATGAAGTGTGATGTGGATATCAGAAAAGATTTGTATGGAAACATTGTTCTCAGTGGTGGTTCTACCATGTTCCCTGGTATCGCTGACCGTATGAGCAAAGAGATCACAGCTCTTGCTCCCAGCAGCATGAAAATCAAGGTCGTTGCACCACCAGAAAGGAAATACAGTGTCTGGATTGGAGGATCAATCCTTGCATCGCTCAGCACCTTCCAGCAGATGTGGATTTCAAAGGGCGAGTACGACGAATCTGGCCCATCCATTGTCCACAGGAAGTGCTTCTAA

ClActin-F0：AGGTGGCAGATGTTTATGATT

ClActin-R1：TTAAGCGTGAATGTAGGGAATAT (rc：ATATTCCCTACATTCACGCTTAA)

ClActin-R0：ATTCGTCGTACTCGCCCTTT(rc：AAAGGGCGAGTACGACGAAT)

ClActin-F1：CTGGGCCTTGTATTACAAAGTGG

ClActin-F0+ClActin-R0=1573 bp

ClActin-F1+ClActin-R1=1464 bp

**[Pumpkin]**

GTCATCCAGGTCACCCTATCTTTACTGGTGCTCCAAATCATTCACTCCAAATTCTTCATTTCATCTGAATTTCATTTACATTTGCATTTCATCCTTTCTTTCCTTTTTTTTTTCTTCTTCCATATAAATATCCTCTTTTTTCCCTTCCCTTCTTCCACTACTTCCTTCTCTCCGTTTTCGATCTCACCTTCTCGCCTTCGCAACCGAGAGAGACTGGGCCTGAAGAAGGGGAGTAGGCCGTCGATTCCTTCACTTTGCTCTCCGATTTTCCTTCCTCTTTCTTGTTTCTTTAGGTATGTTCTCCTTTCGATCTGCTTTTGTTTAGATCTCTTACTCTGCTTTTTATTCGAGATTTGGATTTGCTTTAGCTGCCATTTCTGTTTTTTTTTTTTCAATCACTGTTATGAGCTTCGATCTGGTTTGATTTTTGTCTGTTTTTACCAATGCCTGCCTTGATTTGTGTTGATCAGTACAACTTTTAGCTTCTAGCTCGTGGATTGTATCTTTGTTTAGTGCTTGTTTAGTTCGTTCGTTGTTCTAAGATTTTTTATGCGATTTGTAAATCGGAATGCTGTTTCTTGCTGTACCTGACTCTGTTCTTTTGTTTTTATTCCGTTCTCTCTCTCTCTCTCTTTTTTTTTTTTTTTTTTTTTTTTTTTTTTTTTTTTTTTTTTTTTTTTTTTTTNNNNNNNNNNNNNNNNNNNNNNNNNNNNNNNNNNNNNNNNNNNNNNNNNNNNNNNNNNNNNNNNNNNNNNNNNNNNNNNNNNNNNNNNNNNNNNNNNNNNNNNNNNNNNNNNNNNNNNNNNNNNNNNNNNNNNNNNNNNNNNNNNNNNNNNNNNNNNNNNNNNNNNNNNNNNNNNNNNNNNNNNNNNNNNNNNNNNNNNNNNNNNNNNNNNNNNNNNNNNNNNNNNNNNNNNNNNNNNNNNNNNNNNNNNNNNNNNNNNNNNNNNNNNNNNNNNNNNNNNNNNNNNNNNNTTTTTTTTTTTTTTTTTTTTTTTTTTTTTTTTTTTTTTTTTTTTTTTTTTTTTTTTTTTTTTTTTTTTTGTTCTGTTGTCTTTTTGGAATCTTTTTGATTTGTGAGGAATCGGTGGCCTTTTGATGCGCATTTGCAACCAGTGGCTGTTCTTTGACAATTTACGTGTTTGTTATACAAATTTTATAGTACTGGAGCTGAGATCTTGTGTTTCGTCGAAAGTCGCCTGTAATTTTCCCGATTCAATTTGAATTTTTCTAGCATCGTTTCCATTGCCAATTTTTTACATGTGTTCTGCATGACTGGACTTCTGAAGAAATTTCTATTAAGGATCTAGATTAATTTCCTTGTTCTGTTAATAAGATATGTGGAAAGTTGATGAACTGAGGATCGATTGTTTGCTTACTAGTTAATATAATTTTTAGATTAAAATCGTGCAGCTTACAGTTCTTCCTCGTGTATAAAATTCAACGTTTTTGGCCGTTTTCTGTTTATTTAAGATTTAGACAAAGTTAGAAGTTAGATGGATTTCTAAAGTAGATGTATTATTAGGTGGCAAATATTTTTATATGAACTCTGGGCTTTAAATTACAAGGTGGATTGTCTGTCTTATGCTTACAGCTTACTGACCGTTCAATGTGCATAGCACAGTTGTGCTTTGAATTCTATGTTATATTTTGCTCATCTGTGTCAATTTGTGTGTCTTTCAGTAACTACAAAAGATGGCCGATGCTGAGGATATCCAGCCACTTGTCTGTGACAATGGAACTGGAATGGTGAAGGTTAGATTACTGACCTTAAAATGCAGCTGCAGCTGTTTTTCTTGTTGTAGATCTACCTACCTACGTGCTCAACAATTATTACTGTTTTTCTCAGTATGGTCTTCTTTTGTCTTCACTTTGTTAATTGGCCTGAAGTTTTAAAATTTTATTTTTAGGCTGGATTTGCTGGTGATGATGCTCCTAGGGCAGTGTTTCCCAGCATTGTTGGTCGACCCCGACACACTGGTGTCATGGTAGGTATGGGCCAGAAGGATGCTTATGTCGGTGATGAGGCCCAATCCAAAAGAGGTATTCTCACCTTGAAATATCCCATTGAGCATGGTATTGTGAGCAACTGGGATGATATGGAAAAGATTTGGCATCACACATTCTACAATGAGCTTCGTGTTGCACCTGAAGAACACCCTGTGCTTCTTACTGAAGCACCTCTCAATCCCAAAGCTAACAGAGAAAAGATGACACAAATCATGTTTGAAACTTTTAATGTACCTGCTATGTATGTTGCTATCCAGGCCGTTCTATCTCTGTATGCCAGTGGTCGTACAACAGGTTCACATCCATCTTCTCCTTCTGTCTTAAAGTTTGGACTCTTGGACTTTGATTTGGATAGTATTTTTAATGCATTCTTTGAACAGGTATTGTGCTGGACTCTGGTGATGGTGTTAGTCACACTGTGCCAATCTACGAGGGTTATGCTCTACCCCATGCTATCCTCCGTCTGGACCTTGCTGGTCGTGATCTAACTGATGCCTTGATGAAGATTCTCACTGAGAGAGGGTACATGTTCACAACCACTGCTGAACGGGAAATTGTCCGTGACATGAAAGAAAAGCTTGCTTATGTTGCACTCGACTATGAGCAGGAACTCGAGACTGCTAAGAGTAGCTCCTCAATTGAGAAGAACTACGAACTGCCTGATGGGCAAGTCATCACAATCGGAGCTGAGAGATTCCGTTGCCCTGAAGTTCTTTTCCAGCCATCTCTCATTGGAATGGAAGCTGCTGGAATCCATGAGACTACCTACAACTCAATCATGAAGTGTGATGTGGATATCAGAAAAGATTTGTATGGTAACATTGTTCTCAGTGGTGGTTCTACCATGTTCCCTGGTATTGCAGACCGAATGAGCAAAGAGATCACAGCTCTTGCTCCAAGCAGCATGAAAATTAAGGTCGTTGCACCTCCTGAAAGGAAATACAGTGTCTGGATTGGAGGGTCAATCCTTGCATCGCTCAGCACCTTCCAGCAGGTGAAATACTCCCTACATTCACTCTTAACCTCTCATTTATTCCCATTTCTAGCTATATAGTTTCACACTGATTAATGTAAATACTTCATTTTTGCCTACCAGATGTGGATTTCGAAGGGTGAGTATGATGAATCTGGTCCATCCATCGTCCACAGGAAGTGCTTCTAA

**CDS squnence**

ATGGCCGATGCTGAGGATATCCAGCCACTTGTCTGTGACAATGGAACTGGAATGGTGAAGGCTGGATTTGCTGGTGATGATGCTCCTAGGGCAGTGTTTCCCAGCATTGTTGGTCGACCCCGACACACTGGTGTCATGGTAGGTATGGGCCAGAAGGATGCTTATGTCGGTGATGAGGCCCAATCCAAAAGAGGTATTCTCACCTTGAAATATCCCATTGAGCATGGTATTGTGAGCAACTGGGATGATATGGAAAAGATTTGGCATCACACATTCTACAATGAGCTTCGTGTTGCACCTGAAGAACACCCTGTGCTTCTTACTGAAGCACCTCTCAATCCCAAAGCTAACAGAGAAAAGATGACACAAATCATGTTTGAAACTTTTAATGTACCTGCTATGTATGTTGCTATCCAGGCCGTTCTATCTCTGTATGCCAGTGGTCGTACAACAGGTATTGTGCTGGACTCTGGTGATGGTGTTAGTCACACTGTGCCAATCTACGAGGGTTATGCTCTACCCCATGCTATCCTCCGTCTGGACCTTGCTGGTCGTGATCTAACTGATGCCTTGATGAAGATTCTCACTGAGAGAGGGTACATGTTCACAACCACTGCTGAACGGGAAATTGTCCGTGACATGAAAGAAAAGCTTGCTTATGTTGCACTCGACTATGAGCAGGAACTCGAGACTGCTAAGAGTAGCTCCTCAATTGAGAAGAACTACGAACTGCCTGATGGGCAAGTCATCACAATCGGAGCTGAGAGATTCCGTTGCCCTGAAGTTCTTTTCCAGCCATCTCTCATTGGAATGGAAGCTGCTGGAATCCATGAGACTACCTACAACTCAATCATGAAGTGTGATGTGGATATCAGAAAAGATTTGTATGGTAACATTGTTCTCAGTGGTGGTTCTACCATGTTCCCTGGTATTGCAGACCGAATGAGCAAAGAGATCACAGCTCTTGCTCCAAGCAGCATGAAAATTAAGGTCGTTGCACCTCCTGAAAGGAAATACAGTGTCTGGATTGGAGGGTCAATCCTTGCATCGCTCAGCACCTTCCAGCAGATGTGGATTTCGAAGGGTGAGTATGATGAATCTGGTCCATCCATCGTCCACAGGAAGTGCTTCTAA

CmActin-F0：CGTCGAAAGTCGCCTGTAAT

CmActin-R0：AAGCACTTCCTGTGGACGAT(rc：ATCGTCCACAGGAAGTGCTT)

CmActin-F0+-R0=1982 bp

CmActinF1：TCTAAAGTAGATGTATTATTAGGTGGCA

CmActinR1：ACCAGATTCATCATACTCACCCTTC (rc：GAAGGGTGAGTATGATGAATCTGGT)

CmActin-F1+-R1=1639 bp
