# Supplementary material for: Molecular detection of watermelon virus disease in Liaoning Province, China, and resistance evaluation of nine rootstocks to cucumber green mottle mosaic virus
Source: PeerJ. 2026 Aug 10;14:e21603. doi: 10.7717/peerj.21603 (PMC13471747; doi:10.7717/peerj.21603)
Supplement: Supplementary file 2 [file peerj-14-21603-s022.docx]

**Fig. S1** Gel image of RT-PCR test results for samples from Yingkou City. (A, B) Gaizhou County (Huangdazhai Village and Shanghezhai Village); (C) Lutun Town. M：2000 bp Marker; 1: CGMMV; 2: CMV; 3: WMV; 4: TMV; 5: ZYMV; 6: MYSV; 7: WSMoV; 8: Negative control (ddH2O).

**Fig. S2** Gel image of RT-PCR test results for samples from Xinmin City. (A, B) Liangshan (Wei Hao Village and Jianjiawobao Village); (C) Lujiatun Town; (D) Yaopu Town. M: 2000 bp Marker; 1: CGMMV; 2: CMV; 3: WMV; 4: TMV; 5: ZYMV; 6: MYSV; 7: WSMoV; 8:Negative control (ddH_2_O).

**Fig. S3** Gel image of RT-PCR test results for samples from Chaoyang City. (A) Heishui (Dongtai Village, Huangzhuzi Village); (B) Yichenggong (Fangshen Village, Huoshidi Village). M: 2000 bp Marker; 1: CGMMV; 2: CMV; 3: WMV; 4: TMV; 5: ZYMV; 6: MYSV; 7: WSMoV; 8: Negative control (ddH_2_O).

**Fig. S4** Specificity detection of *Actin* primers in six rootstocks. M: 2000 bp DNA Marker; 1~2: Jingxin Zhenwang; 3~4: Jingxin Zhansheng; 5~6: Yongzhen; 7~8: Jingxin Zhen No. 2; 9~10: Jingxin Zhen No. 4; 11~12: Jingxin Zhen No. 9.

**Fig. S5** Resistance assessment of three additional rootstocks to CGMMV. (A) Phenotypic responses of three watermelon rootstocks (RW1, RW2, RW3) and one representative gourd rootstock (RG3) to CGMMV infection. Upper panels: whole-plant morphology; middle panels: leaf symptoms at 14 days post-inoculation (dpi); lower panels: leaf symptoms at 21 dpi. Control: mock-inoculated negative control plants; +CGMMV: plants mechanically inoculated with CGMMV. Scale bars = 5 cm. (B) Relative CGMMV accumulation levels in nine rootstocks at 21 dpi, quantified by quantitative real-time polymerase chain reaction (qRT-PCR). Different lowercase letters above bars indicate statistically significant differences at P < 0.05 as determined by Duncan's multiple range test. Data are presented as mean ± standard error (SE) from three independent biological replicates.
